# Oral fibrolipoma

**DOI:** 10.4322/acr.2023.431

**Published:** 2023-05-08

**Authors:** Hela Zouaghi, Abdellatif Chokri, Adel Bouguezzi, Nouha Ben Abdeljelil, Sameh Sioud, Hajer Hentati, Jamil Selmi

**Affiliations:** 1 University of Monastir, Faculty of Dental Medicine, Monastir, Tunisia; 2 Research Laboratory Oral Health and Orofacial Rehabilitation, Monastir, Tunisia; 3 Fattouma Bourguiba University Hospital, Department of Pathology, Monastir, Tunisia

**Keywords:** Lipoma, Mouth Neoplasms, Diagnosis

Lipoma is a benign mesenchymal tumor of adipocytes.^1^ It is most common in mid-aged adults with no gender predilection.^2^ It is uncommon in the oral cavity and represents 1 to 4% of all benign lesions in this area. Oral lipoma was first described by Roux in 1848 as “yellow epulis”.^3^ Clinically, it presents as a painless, soft, slow-growing mass covered by normal mucosa that may be yellowish depending on its depth. It can be pedunculated or sessile.^4^ Histopathologically, several subtypes were described, such as angiomyolipomas, myelolipomas, fibrolipomas, ossifying lipoma, hibernomas, spindle cell lipomas, pleomorphic lipomas, chondroid lipomas, and neural fibrolipomas.^5^ Fibrolipomas count as 1.6% of all lipomas.^3^ The mean diameter in the oral cavity is 2 cm, frequently causing esthetic deformity, chewing, and speaking problems.^3^ The treatment is surgical excision. With complete excision of the capsule, the recurrence rate is very low.^5^

The etiology of lipomas remains unclear. Some hypotheses suggested that trauma and inflammation stimulate preadipocyte differentiation and maturation. Genetic factors were also observed in a few cases, with familial hereditary forms of multiple lesions and chromosomal aberration in 12q, 13q, and 6p chromosomes in other cases. Other studies proposed endocrine imbalance in the pathogenesis of fibrolipoma and was linked to diabetes, hormonal therapy, and lipid metabolism disorders.^1,3,5^

Typically, the diagnosis of oral lipoma is made clinically and does not require radiographic examination. However, soft tissue radiography is mandatory in case of pain, rapid growth, giant size, or fixation to the surrounding tissues.^1^ Magnetic resonance imaging (MRI) has been reported to be the most required imaging technique for diagnosing lipomas; they display high signal intensity and appear to be well-encapsulated masses on both T1- and T2-weighted images.^6^ Differential diagnosis includes herniated buccal fat, fibroma, neurofibroma, dermoid cyst, benign salivary gland tumor, hibernomas, angiolipomas, and liposarcoma.^4^ Rare cases of intramuscular and intraosseous lipomas were reported.^5^ Multiple lipomas can be a manifestation of syndromes like multiple familial lipomatosis, benign symmetric lipomatosis (Madelung disease), Gardner syndrome, and adiposis dolorosa.^1^

Histopathologically, the fibrolipoma variant is characterized by focally increased fibrous tissue intermixed with lobules of mature adipose cells.^5^

In immunohistochemistry, it shows vimentin positivity. Also, higher Ki-67 antibody expression in fibrolipoma compared to other lipoma variants is indicative of higher proliferative activity and thus related to higher recurrence and malignant transformation. Consequently, fibrolipoma requires a more meticulous follow-up.^4^

**Figure 1:**
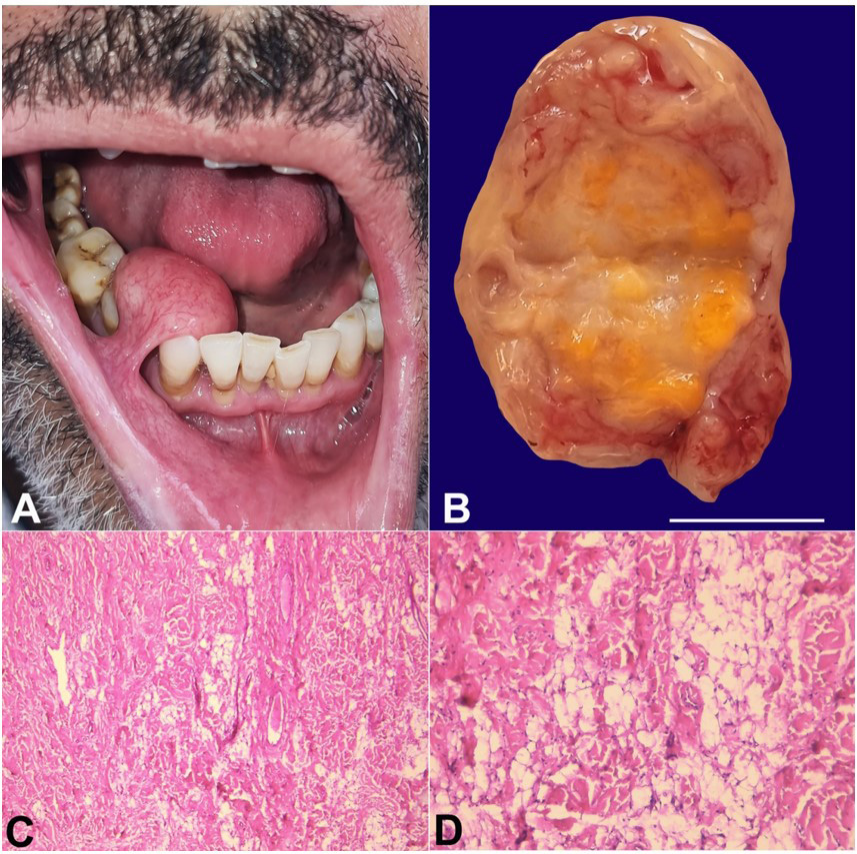
Oral fibrolipoma, **A -** intraoral view of a 2 cm pedunculated nodule on the right buccal mucosa; **B -** macroscopic view of the excised specimen cut in the middle (scale bar= 1 cm); **C** and **D -** mature adipose proliferation associated with paucicellular fibrous tissue (C: H&E x100; D: H&Ex400).

Figure 1 refers to a 49-year-old man referred to the oral surgery department for a buccal nodule that has been evolving for 17 years. He reported slight discomfort in chewing, slow growth of the mass, and no pain. Personal and family histories were noncontributory. Clinical examination revealed a 2 cm pedunculated, pink-yellow, soft nodule on the right buccal mucosa (Figure 1A). This typical presentation was sufficient to suspect the diagnosis of lipoma, and radiographic examination was not required. The mass was wholly removed surgically (Figure 1B). The histological examination showed mature adipose proliferation associated with paucicellular fibrous tissue, consistent with a fibrolipoma (Figure 1C-1D). The patient remains under regular follow-up.
